# Microwave-activated Ni/carbon catalysts for highly selective hydrogenation of nitrobenzene to cyclohexylamine

**DOI:** 10.1038/s41598-017-02519-0

**Published:** 2017-06-01

**Authors:** Xinhuan Lu, Jie He, Run Jing, Peipei Tao, Renfeng Nie, Dan Zhou, Qinghua Xia

**Affiliations:** 1 Hubei Collaborative Innovation Center for Advanced Organic Chemical Materials, Hubei University, Wuhan, 430062 P. R. China; 2 Ministry-of-Education Key Laboratory for the Synthesis and Application of Organic Functional Molecules, School of Chemistry and Chemical Engineering, Hubei University, Wuhan, 430062 P. R. China

## Abstract

Biocarbon supported Ni catalysts have been prepared by facile impregnation of Ni species by microwave-heating and used for selective hydrogenation of nitrobenzene to cyclohexylamine. These catalysts were characterized by X-ray diffraction, Raman spectra, N2 sorption measurement, X-ray photoelectron spectroscopy, temperature programmed reduction of H2 and H2 temperature-programmed desorption. The morphology and particle size of catalysts were imaged by scanning electron microscope and transmission electron microscope. For the hydrogenation of nitrobenzene to cyclohexylamine, 10%Ni/CSC-II(b) exhibits the best catalytic activity to achieve 100 mol% conversion of nitrobenzene and 96.7% selectivity of cyclohexylamine under reaction conditions of 2.0 MPa H2 and 200 °C, ascribed to high dispersion of Ni species and formation of nanosized Ni particles on the support aided by microwave-heating. Thus-prepared Ni/CSC catalyst is greatly activated, in which the addition of precious metal like Rh is totally avoided.

## Introduction

At present, the focus on the environment and the reduction of energy consumption and chemical waste in chemical transformations is a great challenge^1^. Cyclohexylamine (CHA) can be used in the synthesis of artificial sweeteners (sodium or calcium cyclamate), metal corrosion inhibitors, rubber vulcanizing additives, dyestuff, plasticizers and extracting agents for natural products^2^. In general, industrial preparation of CHA is carried out by two steps: i) hydrogenation of nitrobenzene (NB) to aniline^3–10^, ii) hydrogenation of aniline to CHA^11–15^, which is a complicated process. Unfortunately, the CHA selectivity prepared in the above process is generally less than 50%. The products always include dicyclohexylamine (DCHA), diphenylamine, phenylcyclohexylamine, as well as cracked ammonia, cyclohexane and benzene beside cyclohexylamine (CHA)^16^.

Catalytic hydrogenation over the supported transition metals represents a clean alternative but the formation of toxic azo-^17^ and azoxy-^18^ derivatives is still a big drawback. Metal catalysts have been relatively efficient for catalytic hydrogenation of nitro compounds, inclusive of Raney nickel, copper chromite or noble metals including platinum, palladium and rhodium. Although these metals exhibit high catalytic hydrogenation activity, the selectivity to the desired aromatic amines is generally not high. Therefore, it is very important to study the efficient catalyst for highly selective hydrogenation of nitroaromatics, for which there have been several reports on gas-phase continuous conversion of nitrobenzene over Cu/SiO_2_ and Pt/ZSM-5^19, 20^. However, the catalysts containing precious metals are expensive, so that the development of cheap hydrogenation catalysts containing non-precious metals has become the goal of researchers. Many methods have been used to develop environmentally-friendly and economical hydrogenation catalysts and processes, such as catalytic hydrogenation^21–34^, catalytic transfer hydrogenation^35–41^, CO/H_2_O conditions^42–44^ and other reduction systems^45–51^. However, the problem remains unresolved. Our previous work reported highly selective ‘one-step’ catalytic hydrogenation from nitrobenzene to cyclohexylamine over the supported %Ni-‰Rh composite catalysts^52^, in which the addition of 3‰Rh was necessary for enhanced activity of Ni/CSC catalysts. Such a process can completely skip the synthesis, separation and transformation of aromatic amine intermediates, and meet the requirements of energy-saving green chemistry.

**Figure 1:**
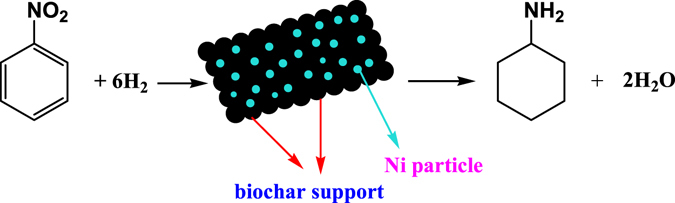
The hydrogenated products of nitrobenzene.

In this work, the Ni/CSC catalysts prepared through supporting cheap Ni species on ball-milled biocarbon by microwave-heating is greatly activated, which is encouraging. A direct synthesis of alicyclic amines from the hydrogenation of nitroarenes over biochar supported non-precious metal Ni catalysts is presented in Fig. 1. In the context, the corresponding effects are deeply revealed by wide reactions and structural characterizations.

## Results and Discussion

### Structural characterizations of Ni/carbon catalysts

**Figure 2:**
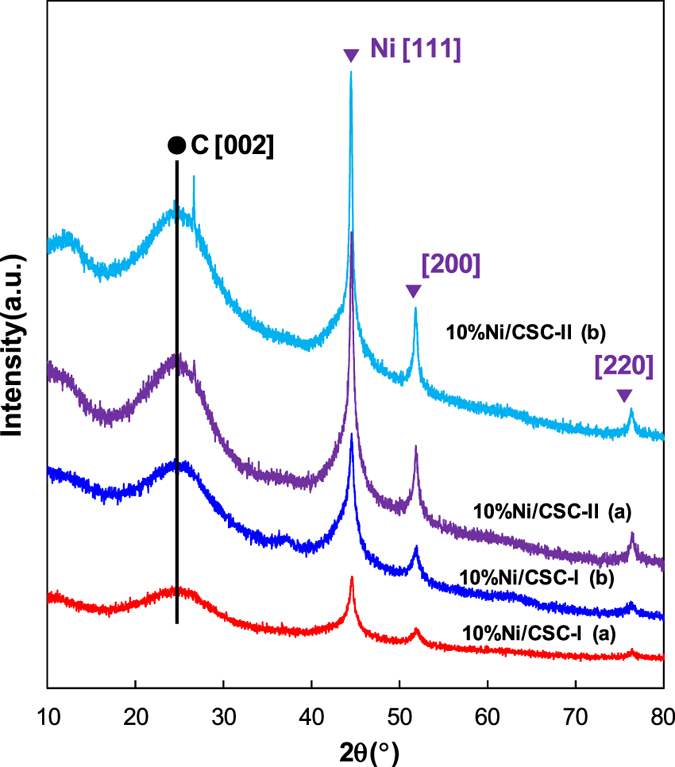
XRD patterns of various 10%Ni/CSC catalysts with different preparation modes.

The XRD patterns of various 10%Ni/CSC catalysts with different preparation modes are shown in Fig. 2. The characteristic peaks of Ni metal (2θ = 44.3, 51.6 and 75.7°) are observed for all the 10%Ni/CSC (coconut shell charcoal) catalysts with different preparation modes, in which three peaks at 44.4°, 51.6° and 76.2° are attributed to [111], [200] and [220] diffraction peaks of Ni^0^. This means that the structure of the CSC has not been destroyed and the majority of NiO is reduced to Ni metal during the preparation. Figure S1 presents the XRD patterns of the samples with different Ni contents. Similarly, the characteristic peaks of CSC (2θ = 23.5°) and Ni metal (2θ = 44.4°, 51.6° and 76.2°) are observed for all the Ni/CSC catalysts with the Ni loading from 3−15 wt%, in which the intensity of [111], [200] and [220] diffraction peaks due to Ni^0^ is gradually increased with increasing the Ni content.

Apparently, SEM images of 10%Ni/CSC-I(a) and 10%Ni/CSC-II(b) exhibit irregular spherical morphologies and high dispersion of Ni component on the CSC (Figure S2). The morphology of catalyst prepared via supporting Ni species on ball-milled CSC by microwave-heating preparation is obviously different from that by the conventional method. The small particles and open pores of 10%Ni/CSC-II(b) catalyst can be seen obviously. Microwave as a kind of electromagnetic wave has a special electric field effect and rapid heating characteristics, can be absorbed by some dielectric materials (such as water) to generate heat, so microwave obtains a wide range of applications in industry, scientific research and other fields^53^. Microwave heating characteristics is to generate heat at different depths of the material simultaneously, and to provide a much uniform heating compared to convebtional heating methods such as ovens (heated by external heat source through the heat radiation from the outside and inside), this improves the quality of the heating greatly.

**Figure 3:**
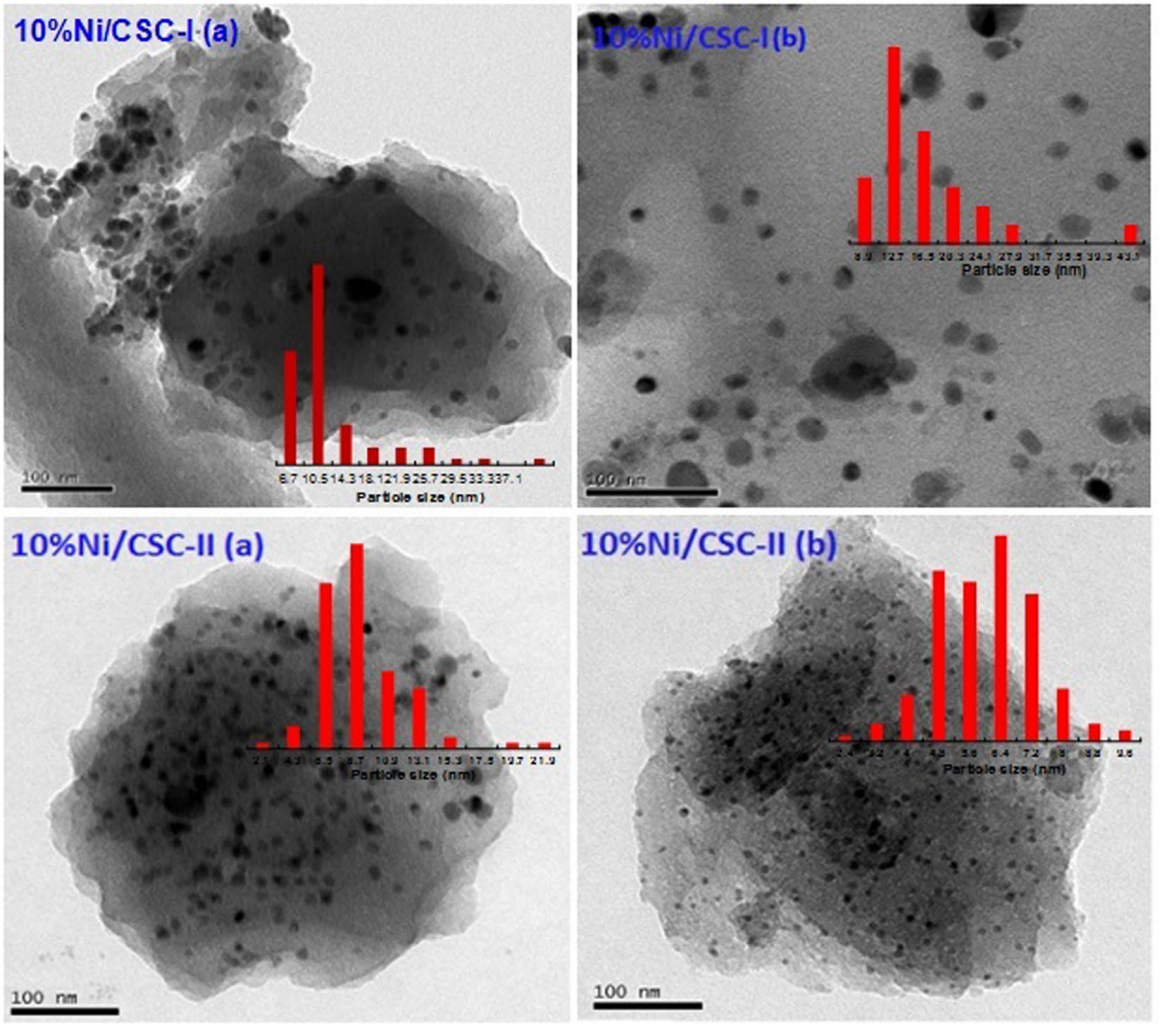
The TEM images of 10%Ni/CSC-I and 10%Ni/CSC-II.

From TEM images in Fig. 3, it is found that the mean particle size of Ni on unmilled CSC is 13.2 nm (10%Ni/CSC-I(a)), similar to that on ball-milled CSC (10%Ni/CSC-I(b)). TEM images of 10%Ni/CSC-II(a) and 10Ni/CSC-II(b) show the presence of uniformly well-dispersed Ni nanoparticles (NPs) on the CSC, in which those NPs exhibit no aggregation into large clusters. The average diameter of Ni NPs on the catalyst 10%Ni/CSC-II(a) is 9.4 nm, distinctly smaller than those of above two samples. However, the size of Ni NPs on 10%Ni/CSC-II(b) is only 5.0 nm, quite smaller than that of 10%Ni/CSC-II(a), which means that microwave-heating is considerably effective for the size control of Ni NPs on the support.

**Figure 4:**
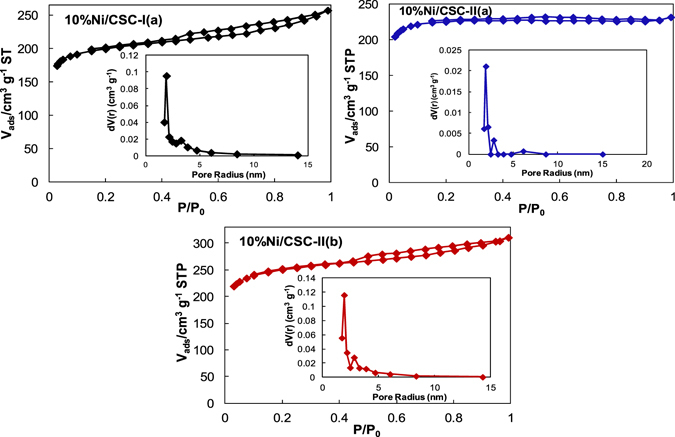
Nitrogen sorption isotherms and pore size distribution of 10%Ni/CSC catalysts.

N_2_ sorption isotherms (Fig. 4) shows steep uptakes at high p/p° relative pressure and a clear hysteresis loop presumed to be related to outer surface features and the presence of micropore domains. In Table 1, BET surface area of 10%Ni/CSC-I(a) is 599.6 m^2^/g, smaller than 678.2 m^2^/g of 10%Ni/CSC-II(a) and 725.9 m^2^/g of 10%Ni/CSC-II(b), with an average pore size of 19.1−19.2 nm for three samples. It is apparent that BET surface area of the 10%Ni/CSC-II(b) catalyst prepared via supporting Ni species on ball-milled CSC by microwave-heating is significantly increased.

**Table 1:** Physical properties of the supported Ni/carbon materials.

Catalysts	Preparation mode	Surface area (m^2^/g)	Particle size (nm)		Relative atomic percentage (%)		
			XRD	TEM	Ni^0^	NiO	Ni(OH)_2_
10%Ni/CSC-I (a)	not ball milled, not microwave heated	599.6	15.5	13.2	9.7	6.9	83.4
10%Ni/CSC-II (a)	not ball milled, microwave heated	678.2	8.9	9.4	12.4	20.0	67.6
10%Ni/CSC-I (b)	ball milling, not microwave heated	623.8	19.1	13.3	—	—	—
10%Ni/CSC-II (b)	ball milled, microwave heated	725.9	6.0	5.0	12.8	37.0	50.2

**Figure 5:**
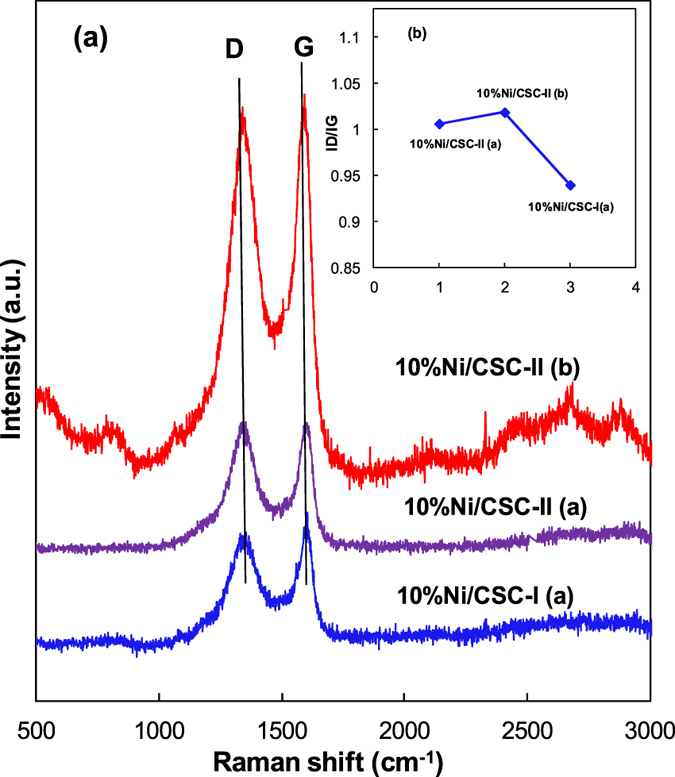
(**a**) Raman spectra and (**b**) ID/IG values of 10%Ni/CSC.

Raman spectra of the catalysts prepared by different preparation modes are shown in Fig. 5, in which the G band at ~1595 cm^−1^ indicates the in-plane vibration of sp^2^ carbon atoms^54^, while the D band at ~1350 cm^−1^ is a defect-induced Raman feature representing the imperfect crystalline structure of the material^55^. The ID/IG value of 10%Ni/CSC-I(a) prepared by conventional impregnation method is 0.94, increased to 1.0 for 10Ni%/CSC-II(a) by microwave heating, and further to 1.02 10Ni%/CSC-II(b) by ball milling and microwave heating. This result indicates the formation of much more structural defects in 10%Ni/CSC-II (b). The results show that the catalyst 10%Ni/CSC-II (b) prepared via supporting Ni species on ball-milled CSC by microwave-heating contains more structural defects and therefore exhibits higher catalytic activity than other catalysts.

**Figure 6:**
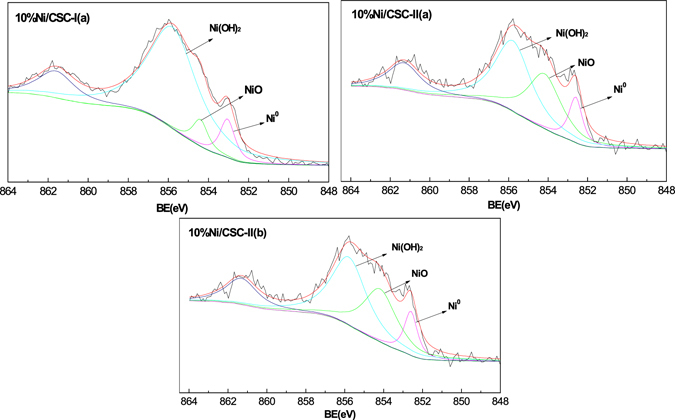
Ni 2p XPS spectra of 10%Ni/CSC-I(**a**), 10%Ni/CSC-II(**a**) and 10%Ni/CSC-II(**b**).

The elemental composition and chemical states of Ni-based catalysts are further determined by XPS analysis. The elemental composition information of all Ni-based catalysts is listed in Table 1. Ni^0^ 2p_3/2_ is observed at 848.1 eV, as shown in Fig. 6. The spectrum of Ni 2p_3/2_ contains the elemental Ni 2p_3/2_ peak at 848.1 eV close to the previously-reported values^56^. The presence of NiO and Ni(OH)_2_ are confirmed by fitting the high-energy shoulders on the metallic Ni line at energies of about 854.2 and 856.0 eV^57^. The presence of NiO species can be attributed in the form of the oxidized Ni state by exposure material in the air after reduction treatment^34, 58^. The percentage of Ni^0^ is 9.7% for 10%Ni/CSC-I(a) prepared by ordinary wet impregnation method, which is increased to 12.4% for 10%Ni/CSC-II(a) and further increased to 12.8% for 10%Ni/CSC-II(b). And the percentage of NiO shows the following descending order: 10%Ni/CSC-I(a) (6.9%) < 10%Ni/CSC-II(a) (20.0%) < 10%Ni/CSC-II(b) (37.0%). For 10%Ni/CSC-II(b) sample, relative concentration of nickel in three different chemical states is 12.8, 37.0 and 50.2% for metallic Ni, NiO and Ni(OH)_2_, respectively. Taking into account that these concentrations are only related to the outer layers of the NPs, these results suggest the formation of a core–shell structure where a pure nickel core is surrounded by a shell of NiO + Ni(OH)_2_. Combined with TPR and TEM characterizations, 10%Ni/CSC-II(b) catalyst has the lowest initial reduction temperature and the smallest particle size, so it is exposed to air the surface is relatively easy to be oxidized quickly.

**Figure 7:**
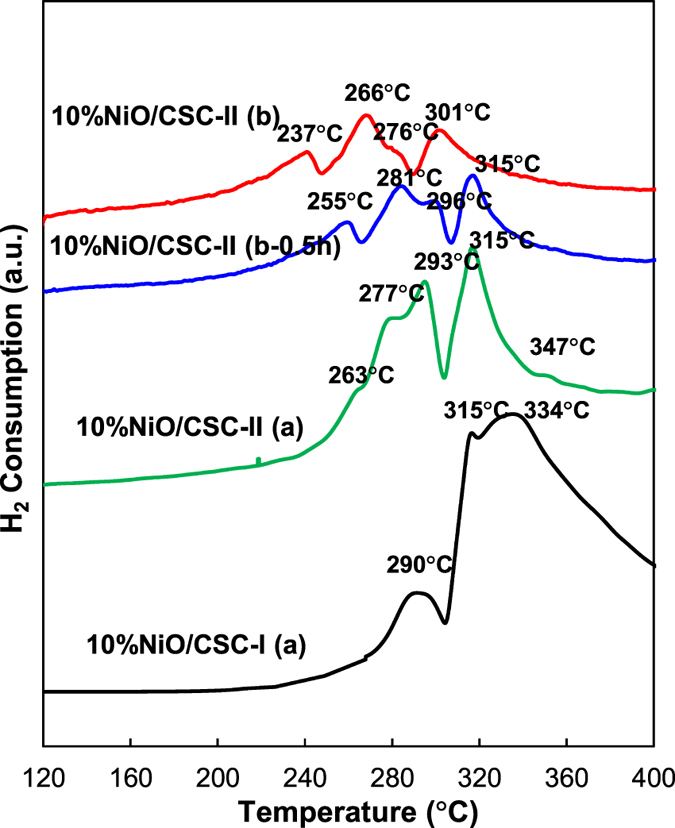
Effect of the catalyst preparation mode on H_2_-TPR profiles of NiO/CSC.

Figure 7 shows the H_2_-TPR profiles of four catalysts, in which the reduction peaks of nickel species appear in the range of 200**−**400 °C. The TPR peaks of NiO dispersed on the support behave as nickel species reduced at low temperature.^51^ The TPR peaks of NiO, which are strongly associated with the carrier, emerge in the region of high temperature^59^. For 10%NiO/CSC-I(a) prepared by conventional impregnation method, the TPR profile has three TPR signals at 290, 315 and 334 °C, ascribable to the reduction of NiO particles to metal Ni0 without interaction with the support^52^, and to the reduction of Ni^2+^ with medium interaction (at 334 °C). For 10%NiO/CSC-II(a), there are two TPR reduction peaks at low temperature of 263 and 277 °C ascribable to the reduction of NiO particles to metal Ni^0^, and one peak at 347 °C attributable to the reduction of Ni^2+^ with strong interaction. For 10%NiO/CSC-II(b), the H_2_-TPR profile shows that the reduction of nickel species on CSC occurs in four stages with maximal signals at 237, 266, 276 and 301 °C, in which the first reduction peak appears at lower temperature than the former three samples. When the microwave-heating time is shortened from 2 to 0.5 h, four reduction peaks in the H_2_-TPR profile of 10%NiO/CSC-II(b-0.5 h) shift to higher temperatures with maximal signals at 255, 281, 296 and 315 °C.

H_2_-TPD experiments were performed to obtain the information of surface structure of the catalysts and to determine the amount of chemisorbed hydrogen quantitatively, in which the latter is a good indication of metal dispersion degree. H_2_-TPD profiles from 282 to 505 °C for 10%Ni/CSC-I(a) and 10%Ni/CSC-II(b) are given in Figure S3, where shows two H_2_ desorption peaks, indicating that hydrogen may be bounded to at least two different Ni surfaces^60^. The comparison of the peak area shows that 10%Ni/CSC-II(b) catalyst has more chemisorbed hydrogen than 10%Ni/CSC-I(a) catalyst, for which the hydrogen absorption amount of the catalyst 10%Ni/CSC-II(b) is 1.49 times that of the catalyst 10%Ni/CSC-I(a) after quantitative calculation. This indicates that Ni dispersion in 10%Ni/CSC-II(b) catalyst is significantly higher than that in the 10%Ni/CSC-I(a) catalyst.

### Catalytic one-step hydrogenation of nitrobenzene

**Figure 8:**
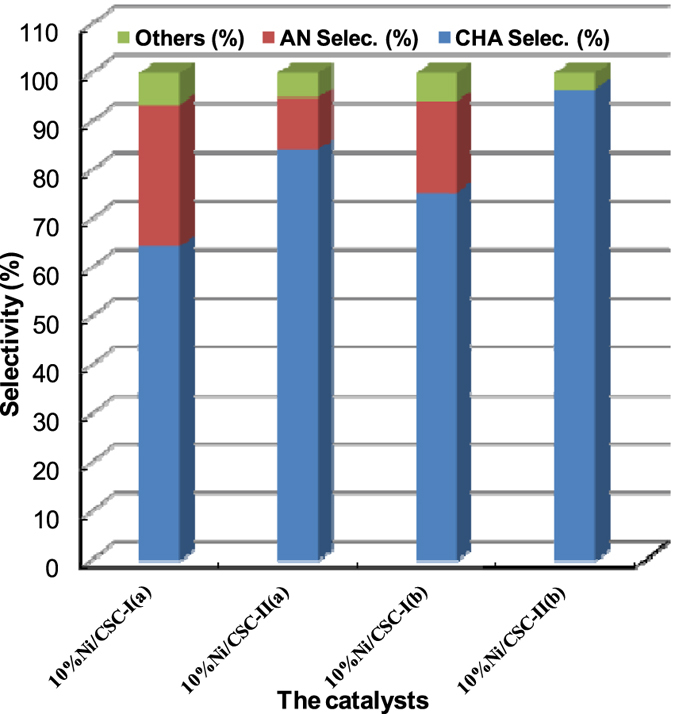
Effect of catalyst preparation modes on the hydrogenation at 4 h.

As mentioned above, microwave-heating can be used as an efficient tool to improve the surface property of the catalyst. Figure 8 compares the activity of the catalysts prepared by traditional mode and by microwave-heating in the catalytic hydrogenation reaction. For the catalyst 10%Ni/CSC-I(a) prepared under traditional mode (magnetic stirring with unmilled CSC), the hydrogenation reaction conversion of NB could reach 100 mol% with only 64.4% selectivity of CHA (fully-hydrogenated product) and the partially-hydrogenated product selectivity of aniline (AN) is 29.0%. Over the catalyst 10%Ni/CSC-II(a) prepared by microwave-heating (unmilled CSC), the hydrogenation reaction conversion of nitrobenzene (NB) is also maintained at 100 mol%, and the selectivity of cyclohexylamine (CHA) can be increased to 84.2%. Noticeably, the selectivity of CHA is 75.5% on 10%Ni/CSC-I(b) (ball-milled CSC/no microwave-heating), which, however, is lifted to 96.7% over 10%Ni/CSC-II(b) prepared via supporting Ni species on ball-milled CSC by microwave-heating. This can be related to several advantages promoted by microwave-heating, such as high dispersion of Ni species on the carrier, easy reduction of surface Ni species, and formation of uniformly nanosized Ni particles.

**Figure 9:**
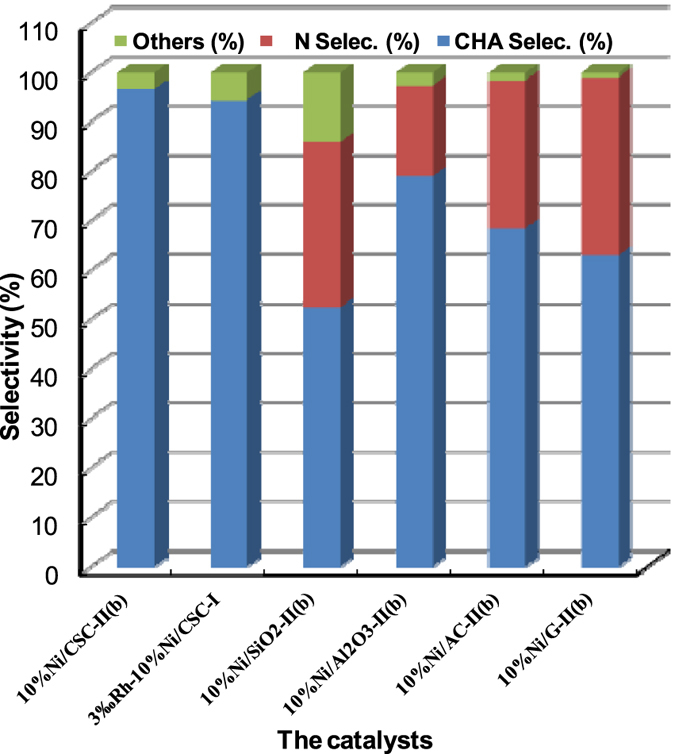
Hydrogenation of NB over different Ni catalysts at 4 h.

As expected, the catalyst 10%Ni/CSC-II(b) achieves the highest CHA selectivity of 96.7% with 100% conversion of substrate under optimal reaction conditions (Fig. 9), even higher than 91.6% selectivity of 3‰Rh-doped 10%Ni/CSC-I prepared by the conventional method^52^. The Ni-based catalysts inclusive of 10%Ni/SiO_2_-II(b) and 10%Ni/Al_2_O_3_-II(b) receive a poor CHA selectivity of < 80%. When other carbon materials such as the activated carbon (AC) and graphite (G) are used as the carriers, the selectivity of CHA is only 68.5% for 10%Ni/AC-II(b) and 63.0% for 10%Ni/G-II(b). Importantly, the 10%Ni/CSC-II(b) catalyst can afford six recycles without an obvious loss in its activity (Figures S4–S6).

**Figure 10:**
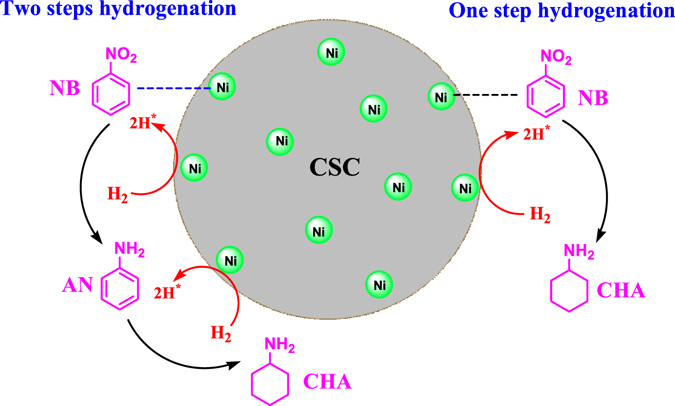
The hydrogenated processes of nitrobenzene.

Table 2 shows the effect of time on the hydrogenation of NB, which shows the induction period characteristic of this 26.8 mol%, the product mixture includes AN (69.0%) and CHA (31.0%). Within 1 h, the conversion of NB is about 89.3 mol%, which is rapidly increased to 100 mol%. The selectivity of fully-hydrogenated CHA is lower than 61% within 1 h, which is increased to 90.5% in 2 h, and to the maximum of 96.7% in 4 h. As the reaction time is prolonged to above 6 h, the selectivity of CHA is slightly decreased to 93.1%. Meanwhile, the hydrogenation of aniline was also carried out under the same reaction conditions (200 °C, 2.0 MPa H_2_). Within 1 h, the conversion of aniline is only 56.2 mol% and the selectivity of CHA was 100%. When the reaction time was prolonged to 4 h, the conversion of aniline was still as high as 100 mol%, but the selectivity of CHA dropped to 91.2%. Therefore, catalytic hydrogenation process of nitrobenzene is not entirely in accordance with the sequence of traditional two-step route, i.e. nitrobenzene is hydrogenated to aniline and then to CHA. The current hydrogenation reaction may proceed with one combination of one-step and two-step processes, as illustrated in Fig. 10.

**Table 2:** Effect of reaction time on the hydrogenation.

Catalyst	Time	Conversion (mol%)	Selectivity (%)		
			AN	CHA	Others
10%Ni/CSC-II(b)	0.5	26.8	69.0	31.0	0.0
	1.0	89.3	42.4	57.6	0
	2.0	100	8.5	90.5	1
	4.0	100	0	96.7	3.3
	6.0	100	0	93.1	6.9

The recycling experiments of 10%Ni/CSC-II catalyst for the hydrogenation of nitrobenzene are conducted. As observed from Figure S4, the decrease in catalyst performance is much milder with successive cycles. Obviously, the conversion has kept steady for 6 recycles with about 96.7% selectivity of CHA. ICP-OES analysis of THF spent solutions did not detect any metal, indicating that leaching was not the cause of the decreased activity. In addition, hot filtration experiments proved that the active phase was not homogeneous.

### Catalytic hydrogenation of other substituted nitro-benzenes

To further demonstrate the versatility of the 10%Ni/CSC-II(b) catalyst, the selective hydrogenation of different substituted nitrobenzenes is investigated. Table 3 summarizes the catalytic hydrogenation results under the same reaction conditions. 3-methyl NB, 2-nitro toluene and 4-methyl NB are mainly converted at 200 °C to the corresponding hydrogenated products with excellent selectivities, in which the full-hydrogenation selectivity is 92.3%, 95.2% and 86.5%, respectively. For chlorine-substituted nitrobenzene (chloro-NB), the maximum selectivity of fully-hydrogenated products does not exceed 81%, possibly relevant to the occurrence of partially-dechlorinated reaction during the hydrogenation process, for which dechlorinated products have been detected by GC-MS. And, reductive dechlorination of chloro-NB to CHA is detected, similar to our previous report^52^. Our studies clearly show that the activation of CSC supported cheap Ni catalyst (10%Ni/CSC-II(b)) by microwave-heating mode for highly selective one-step hydrogenation of nitrobenzene to CHA through tuning the dispersion of Ni species and the size of Ni NPs.

**Table 3:** The hydrogenation results of other substituted nitrobenzenes.

Catalysts	Solvent	Conversion (mol%)	Selectivity (%)		
			Partial	Full	Others
10%Ni/CSC-II(b)		100	0	96.7	3.3
		100	0	92.3	8.8
		100	0	95.2	3.2
		100	0	86.5	13.5
		100	8.6	78.2	13.2
		100	4.8	79.1	16.1
		100	2.9	80.6	16.5

## Conclusions

In this work, biocarbon material CSC supported Ni NPs catalysts have been activated by microwave-heating. The catalytic studies show that 10%Ni/CSC-II(b) containing Ni nanoparticles with the size of 5.0 nm is highly active and stable for the hydrogenation of nitrobenzene to cyclohexylamine with H_2_. The 10%Ni/CSC-II(b) catalyst can afford six recycles without an obvious loss in its activity. Highly catalytic performance can be attributed to high dispersion of Ni species on the carrier, easy reduction of surface Ni species, and formation of uniformly nanosized Ni particles. Combined with TPR and TEM characterizations, 10%Ni/CSC-II(b) catalyst has the lowest initial reduction temperature and the smallest particle size, so it is exposed to air the surface is relatively easy to be oxidized quickly.

## Methods

### Chemicals and Materials

All reagents were obtained from Shanghai Aladdin Chemical Co. Ltd, except that SiO_2_ was purchased from TCI (Shanghai) Development Co. Ltd. Note that CSC is made from high-quality coconut shell biomaterial, which has porous structure, large specific surface area, strong adsorption ability and easy regeneration.

### Preparation of 10%Ni/CSC-I

The supported catalysts 10%Ni/CSC-I(a) is prepared through a conventional wet impregnation method, as reported in our previous article^52^. The 10%Ni/CSC-I(b) catalyst is prepared also through a conventional wet impregnation method similar to 10%Ni/CSC-I(a), in which the support CSC is ball milled in a ball mill at 400 r/min for 2 h.

### Preparation of 10%Ni/carbon-II

The supported %Ni/carbon-II catalysts were prepared through the following procedure. Typically, 1 g of carbon (such as CSC (coconut shell charcoal), AC (activated carbon) and G (graphite)) was ground into fine powders in a ball mill at 400 r/min for 2 h before it was dispersed into the solution consisted of Ni(NO_3_)_2_•6H_2_O (0.162–0.811 g for CSC, 0.541 g for AC and G) and water (40 g). Then, the resulting mixture was heated to 90 °C in a microwave reactor with magnetic stirring. After completion of the reaction, the water was completely evaporated off by a rotary evaporator. The solid was dried in a vacuum oven at 100 °C for 12 h, the resulting powder was transferred into a crucible, heated to 400 °C at a rate of 5 °C•min^−1^ under a flow of hydrogen and kept for 3 h in a tube furnace. The resultant catalyst was designated as *x*%Ni/CSC-II(b), 10%Ni/AC-II(b), 10%Ni/G-II(b), in which *x*% corresponds to 3−15%. Other materials supported metal catalysts were prepared with a similar method, which was designated as 10%Ni/SiO_2_-II(b) and 10%Ni/Al_2_O_3_-II(b). Note that the 10%Ni/CSC-II(a) catalyst was prepared with a similar method except that the carrier CSC was not ball-milled in a ball mill.

### Characterizations

Powder X-ray diffraction (XRD) was performed on a Bruker D8A25 diffractometer with CuKα radiation (λ = 1.54184 Å) operating at 30 kV and 25 mA in the range of 5−80° 2θ. The morphology and size of crystals were imaged with a JEOL JSM-6510A Scanning electron microscope (SEM). Transmission electron microscope (TEM) images were obtained using an accelerating voltage of 200 kV on a JEOL-135 2010F Transmission Electron Microscope. Raman spectra were collected at room temperature from 100 to 4000 cm^−1^ with 514.5-nm argon ion laser (Rhenishaw Instruments, England). N_2_ adsorption was carried out at −196 °C using an auto-adsorption analyzer (Micromeritics, TriStar II). Raman spectra were collected at room temperature from 100 to 4000 cm^−1^ with 514.5 nm argon ion laser (Rhenishaw Instruments, England). The spectra were recorded at a resolution of 2 cm^−1^. The spectra were recorded with a resolution of 2 cm^−1^. X-ray photoelectron spectra (XPS) were recorded on a Perkin-Elmer PHI ESCA system. X-ray source was standard Mg anode (1253.6 eV) at 12 kV and 300 W. The Temperature programmed H2 reduction (TPR) on a TP-5076 dynamic adsorption analyzer, which was carried out in a N_2_ flow of 50 ml/min (10 vol% H_2_), from room temperature to 600 °C at a heating rate of 10 °C/min. H_2_ Temperature-programmed desorption (TPD) of the catalysts was also conducted on a TP-5076 dynamic adsorption analyzer chemisorption instrument. H_2_-TPD was carried out in a stream of argon with a flow rate of 40 mL/min and a temperature ramp of 10 °C/min. Hydrogen consumption was monitored by a thermal conductivity detector (TCD) linked to a computer data acquisition system. The TCD signals were calibrated using 5 μL of H_2_ as standards.

### Catalytic hydrogenation of nitrobenzene

Catalytic tests were conducted in a 100-mL stainless steel autoclave with a magnetic bar. Heterogeneous Ni catalyst 100 mg, 8.0 mmol substrate (nitrobenzene or substituted nitrobenzenes), 100 mg LiOH, and 10 ml solvent tetrahydrofuran (THF) were introduced into the autoclave, which was then vacuumed and purged with H2 three times before it was finally pressurized with 2.0 MPa H_2_ gas. Subsequently, the reaction mixture was stirred (the stirring rate of 250 r/min) at 200 °C for 4 h. After completion of the reaction, the autoclave was cooled down to room temperature, excess H_2_ was carefully released. The resultant product mixtures were analyzed by an GC9720 gas chromatograph with a 30 m capillary column (Rtx@-5) using a flame ionization detector (FID). The hydrogenated products were mainly consisted of aniline (AN), cyclohexylamine (CHA) and others (dicyclohexylamine and some cracked byproducts).

## Electronic supplementary material

Supplementary information
