# Supplementary material for: Microwave-activated Ni/carbon catalysts for highly selective hydrogenation of nitrobenzene to cyclohexylamine
Source: Sci Rep. 2017 Jun 1;7:2676. doi: 10.1038/s41598-017-02519-0 (PMC5453968; doi:10.1038/s41598-017-02519-0)
Supplement: Supplementary file 1 — Supplementary information [file 41598_2017_2519_MOESM1_ESM.doc]

**Microwave-activated Ni/carbon catalysts for highly selective hydrogenation of nitrobenzene to cyclohexylamine**

**Xinhuan Lu^1,2^, Jie He^1,2^, Run Jing^1,2^, Peipei Tao^1,2^, Renfeng Nie^1,2^, Dan Zhou^1,2^, Qinghua Xia^1,2^**

**Figure S1.** XRD patterns of various Ni/CSC catalysts with different Ni contents.


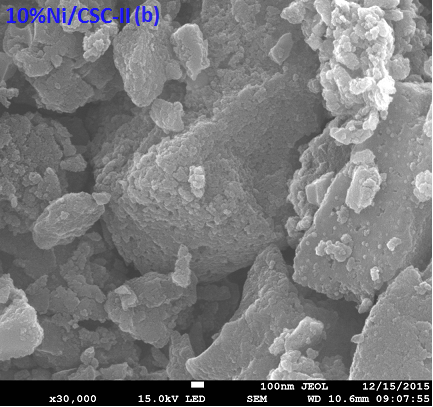

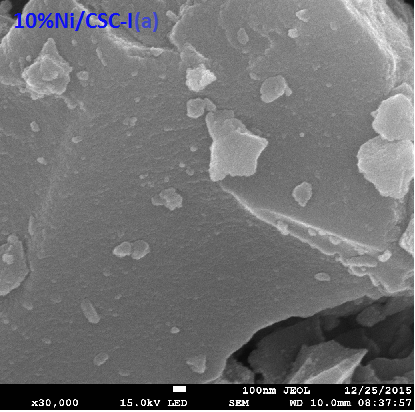


**Figure S2.** SEM-10%Ni/CSC-I(a) and 10%Ni/CSC-II(b)

**Figure S3.** H_2_-TPD profiles of 10%Ni/CSC-I(a) and 10%Ni/CSC-II(b).

**Figure S4.** Recycling results of the catalyst 10%Ni/CSC-II(b).

**Figure S5.** XRD patterns of 10%Ni/CSC-II before and after recycling .

**
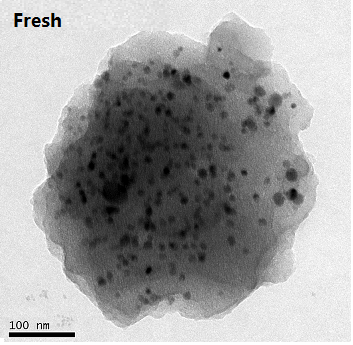

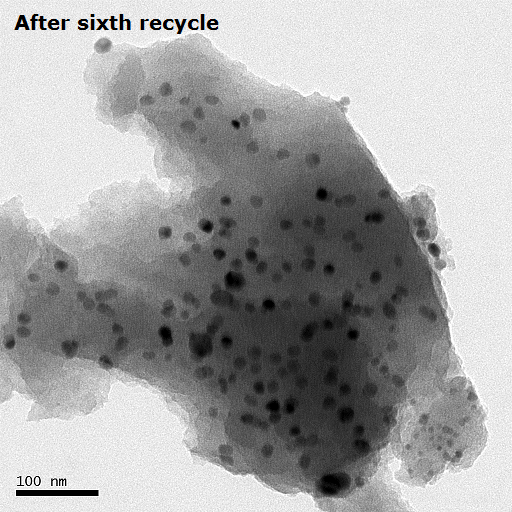
**

**Figure S6.** TEM images of 10%Ni/CSC-II before and after recycling.
